# Store Operated Calcium Entry in Cell Migration and Cancer Metastasis

**DOI:** 10.3390/cells10051246

**Published:** 2021-05-19

**Authors:** Ayat S. Hammad, Khaled Machaca

**Affiliations:** 1 Calcium Signaling Group, Weill Cornell Medicine Qatar, Doha 24144, Qatar; ash2013@qatar-med.cornell.edu; 2 College of Health and Life Science, Hamad bin Khalifa University, Doha 24144, Qatar; 3 Department of Physiology and Biophysics, Weill Cornell Medicine Qatar, Education City, Qatar Foundation, Doha 24144, Qatar

**Keywords:** cell migration, STIM1, orai1, store-operated Ca^2+^ entry, Ca^2+^ signaling, focal adhesions, polarization, cancer, metastasis

## Abstract

Ca^2+^ signaling is ubiquitous in eukaryotic cells and modulates many cellular events including cell migration. Directional cell migration requires the polarization of both signaling and structural elements. This polarization is reflected in various Ca^2+^ signaling pathways that impinge on cell movement. In particular, store-operated Ca^2+^ entry (SOCE) plays important roles in regulating cell movement at both the front and rear of migrating cells. SOCE represents a predominant Ca^2+^ influx pathway in non-excitable cells, which are the primary migrating cells in multicellular organisms. In this review, we summarize the role of Ca^2+^ signaling in cell migration with a focus on SOCE and its diverse functions in migrating cells and cancer metastasis. SOCE has been implicated in regulating focal adhesion turnover in a polarized fashion and the mechanisms involved are beginning to be elucidated. However, SOCE is also involved is other aspects of cell migration with a less well-defined mechanistic understanding. Therefore, much remains to be learned regarding the role and regulation of SOCE in migrating cells.

## 1. Introduction

Cell migration is essential for the development of multicellular organisms and is critical for many physiological processes, including organ development, morphogenesis, tissue repair and homeostasis, immune response and wound healing [1]. It is also essential for tumor metastasis to colonize remote sites, which is the main cause of death from cancers [2]. For adherent cells to move in a directional fashion multiple coordinated processes need to occur, including extension of lamellipodia at the front of the cell, disassembly of focal adhesions at the rear of the migrating cell and force generation through cytoskeleton attachments to the extracellular matrix (ECM) to pull the cell forward. These events require a complex coordinated machinery that involves environmental cues, signaling and cytoskeleton components, as well as focal adhesion remodeling among other mechanisms. Interestingly, many of these aspects are Ca^2+^ dependent, highlighting the critical role of Ca^2+^ signaling in regulating cell movement. In this review, we briefly outline the involvement of Ca^2+^ signaling in cell migration while focusing on the role of store-operated Ca^2+^ entry (SOCE). 

## 2. Cell Migration

**Figure 1:**
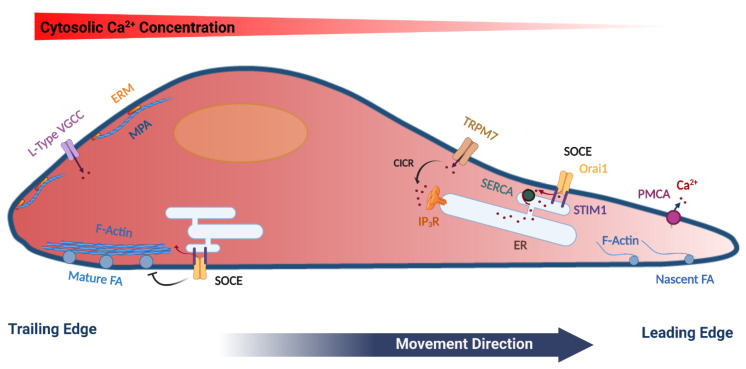
Model summarizing the polarization of various cytoskeletal and Ca^2+^ signaling components in a migrating cell. Mature focal adhesions (FA), L-type voltage-gated Ca2+ channels (VGCC), ERM (Ezrin, Radixin and Moesin) proteins, as well as cortical actin (Membrane Proximal Actin (MPA) are enriched at the rear end of the cell. TRPM7 and the plasma membrane Ca^2+^-ATPase (PMCA) are enriched at the leading edge of the migrating cell. Store-operated Ca^2+^ entry (SOCE), which is mediated by STIM1 and Orai1 have been functionally implicated in disassembly of FA at the rear end, as well as in refilling Ca^2+^ stores at the leading edge. See text for further details. The figure was created using BioRender.com.

Cell migration is a complex coordinated process that incorporates many cellular components and responds to a plethora of environmental cues. Typically, those environmental signals guide the directional migration of cells [1]. For a cell to move in a directional fashion it needs to polarize, with membrane extensions (lamellipodia) at the front end that are later stabilized by nascent focal adhesions. Lamellipodia are driven by actin polymerization followed by attachment to the extracellular matrix (ECM) to allow for force generation. Attachment is mediated by nascent adhesions, which further mature through interaction with the actin cytoskeleton and myosin mediated force generation (Figure 1). At the rear of the cell, mature focal adhesions disassemble to allow the cell body to be pulled forward thus mediating directional cell movement [3]. Focal adhesions are large dynamic plasma membrane-associated macromolecular assemblies that are rich in integrins, and connect the actin cytoskeleton to the extracellular matrix [4]. The dynamic regulation of focal adhesions is essential for successful cell migration and is mediated by Ca^2+^ dependent assembly and disassembly cycles [5,6], as will be further discussed below. 

The actin cytoskeleton is vital in the regulation of focal adhesions as well as in generating the forces required for cell migration. In that context, actin dynamics are regulated by small GTPases, including Rac1, Cdc42 and RhoA [7]. Rac regulates protrusive forces in lamellipodia through modulating actin formation via actin nucleation complexes such as SCAR/WAVE and Arp2/3 [7,8]. Rho, Rho-kinase and Ca^2+^/calmodulin-activated myosin-light chain kinase regulate actomyosin fibers contraction that cause the retraction of the trailing end of the cell and its net forward movement [7,9]. Cdc42 regulates cell polarity in migrating cells through interactions with the PAR complex and the actin cytoskeleton [10,11].

Therefore, the actin cytoskeleton through spatially controlled cycles of polymerization and depolymerization supports membrane deformation and force generation that are required for cell movement. In addition to the actin fibers that crisscross the cell, cells form a shell of cortical actin bundles around their periphery. Cortical actin directly interacts with the PM through the ERM proteins (ezrin, radixin and moesin), that associate with PtdIns (4,5) P2 at the cell membrane through their FERM domain at one end and with actin at the other end, thus linking the PM to the actin cytoskeleton [12]. In addition, cortical actin counters the internal cellular pressure to regulate membrane tension, thus preventing non-specific membrane deformation (blebbing), which impinges on cell migration [13]. The distribution of ERM proteins in migrating cells is polarized with enrichment in the rear of the cell [14] (Figure 1). In contrast, at the front of migrating cells actin is dynamically remodeled through actin severing, nucleation and contraction [15]. Recently, Bisaria et al. developed novel reporters to visualize cortical actin dynamics and more specifically the outer most layer of cortical actin that links directly to the PM, to which they refer as membrane proximal actin (MPA). They found a gradient of MPA density across the migrating cell with high MPA at the trailing edge and membrane protrusions generated from areas with low MPA density [16] (Figure 1). This low density of MPA is maintained by high cofilin activity in the front of the cell, which provides free G-actin monomers to initiate protrusions and decrease MPA density to allow for lamellipodia extension [16,17].

## 3. Ca^2+^ Signaling

Cytoplasmic Ca^2+^ transients that underlie cellular responses can be mediated either by Ca^2+^ release from intracellular Ca^2+^ stores (primarily the ER), or Ca^2+^ entry from the extracellular environment [18]. Physiologically Ca^2+^ transients are typically generated in response to activation of cell surface receptors (G-protein or tyrosine-kinase coupled receptors) leading to stimulation of PLCs that hydrolyze PI (4,5) P_2_ at the cell membrane producing the second messengers IP_3_ and DAG. IP_3_ diffuses readily and binds IP_3_ receptors (IP_3_R) on the ER membrane, which are ligand gated cation channels, thus releasing Ca^2+^ from ER stores. DAG is a lipid second messenger that diffuses within the plasma membrane (PM) and activate PKC, thus expanding the signaling modalities. DAG and Ca^2+^ combine to activate PKC and by extension its downstream targets to code for specific cellular responses. Interestingly, many receptors activate this signal transduction cascade with disparate cellular responses ranging from cell migration to division. Therefore, somehow Ca^2+^ signals produced through the PLC-IP_3_ pathway must encode specificity to direct a particular cellular response. Much of the specificity is encoded in the spatial and temporal properties of the Ca^2+^ signals produced [19]. Such specificity arises from the combination of Ca^2+^ release from stores in response to agonists, but also, a subsequent Ca^2+^ influx from the extracellular space. Ca^2+^ influx can be mediated through receptor-operated cation channels of the TRP family, which can be activated by DAG or its metabolites among other diverse stimuli [20]. Alternatively, Ca^2+^ release from stores results in Ca^2+^ store depletion leading to activation of store-operated calcium entry (SOCE) [21]. SOCE plays important roles in cell migration as will be discussed in detail in the following sections.

## 4. Polarized Ca^2+^ Signals in Migrating Cells

As is the case with the actin cytoskeleton and its modulators, Ca^2+^ signaling in migrating cells is polarized. This was first reported three decades ago as an ascending front to rear Ca^2+^ gradient across the cell (higher Ca^2+^ at the rear compared to the front) [22,23] (see Figure 1). This gradient appears to be mediated at least in part by localization of the plasma membrane Ca^2+^ ATPase (PMCA) to the front of migrating cells resulting in enhanced Ca^2+^ extrusion out of the cell [24]. In addition, migrating cells exhibit polarized Ca^2+^ influx through voltage-gated L-type Ca^2+^channels at the trailing edge, thus supporting increased Ca^2+^ concentration at the back of migrating cells [9,25]. However, the molecular mechanisms controlling polarized PMCA and L-type Ca^2+^ channel activities are not fully understood. 

Furthermore, localized Ca^2+^ pulses are detected preferentially at the front of the migrating cell and interestingly they require Ca^2+^ influx through the stretch activated TRPM7 channel [26,27] (Figure 1). The amplitude and frequency of these TRPM7-mediated Ca^2+^ transients are also regulated by Ca^2+^ release through IP_3_ receptors as well as the actin cytoskeleton [26]. Ca^2+^ influx through TRPM7 induces Ca^2+^-induced Ca^2+^ release through IP_3_ receptors to further enhance the Ca^2+^ transient and regulate actin dynamics (Figure 1). Furthermore, Ca^2+^ pulses at the front of migrating cells have been implicated in stimulating myosin activity to support the formation of nascent adhesions, which presumably provide the anchor for the traction generated by actino-myosin to support cell movement [28,29]. TRPM7 has been implicated in regulating myosin II-based cellular tension and focal adhesions in cancer cells in a polarized fashion as well [30], arguing that the polarized TRPM7-mediated Ca^2+^ pulses at the front of the cell are responsible for regulating cellular tension. These findings are consistent with the increased PMCA activity at the front of migrating cells resulting in lower basal Ca^2+^ levels that would be expected to inhibit persistent myosin light chain kinase (MLCK) activation and allow small local Ca^2+^ pulses to effectively stimulate actino-myosin contraction [24,31]. 

Furthermore, Ca^2+^ signaling modulates actin polymerization at the leading edge of migrating cells resulting in the formation of protrusive structures such as lamellipodia, filopodia and invadopodia/podosomes that help cell movement [32]. During forward movement, local Ca^2+^ signals at the leading edge play a major role in activating actin-based contraction that regulate lamellipodia retraction and adhesion cycles [29,33]. Ca^2+^ influx at the leading edge is required for actin polarization and PI3K activation [33], as well as regulating translocation and activation of the small GTPase Rac1 [34]. In addition, in migrating neuronal cells Ca^2+^ -through the Ca^2+^ sensitive GTPase scaffolding protein IQGAP1- regulates the Rho-family GTPase CDC42 [35]. Rho GTPases regulate actin dynamics and they are augmented in specific structures during cell migration, such as RhoA around focal adhesion complexes, Rac1 in lamellipodia, and Cdc42 adjacent to filopodia [7,36,37]. Furthermore, Ca^2+^-dependent kinases, such as protein kinase C and Ca^2+^ calmodulin-dependent kinase, modulate actin dynamics [36,37,38]. Finally, Ca^2+^ signaling can also modulate the activity of the F-actin severing protein cofilin and myosin, thus affecting cell migration [29,37,39]. 

Collectively these studies show that polarized Ca^2+^ signaling in migrating cells is important to support cell migration by spatially and temporally regulating the actin cytoskeleton. 

## 5. Store-Operated Ca^2+^ Entry (SOCE)

**Figure 2:**
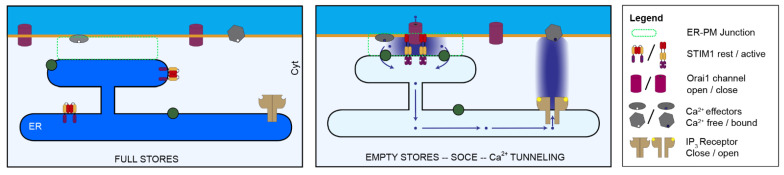
SOCE and Ca^2+^ Tunneling. SOCE is activated in response to a decrease in Ca^2+^ levels in the ER following Ca^2+^ release. This leads to a conformational change in STIM1 and its translocation to ER-PM junctions where it recruits and gates Orai1 allowing Ca^2+^ influx into the SOCE microdomain. During Ca^2+^ signaling in response to agonist with open IP_3_ receptors, Ca^2+^ flowing through Orai1 is taken up by SERCA at ER-PM junctions and diffuses to open IP_3_ receptors that are distant from the SOCE microdomain thus allowing Ca^2+^ release to activate distal effectors.

SOCE represents a primary Ca^2+^ influx pathway in non-excitable cells, which are typically the mobile cells in multicellular organisms. SOCE is activated in response to depletion of intracellular Ca^2+^ stores and is mediated by the STIM and Orai protein families [21] (Figure 2). Interestingly, before being implicated in SOCE, STIM1 was initially cloned as a candidate tumor suppressor gene on chromosome 11 region p15.5 and named GOK [40,41]. Deletions in the 11p15.5 region are associated with childhood cancers, including rhabdoid and Wilms’ tumor [39,40,42,43]. Independently, STIM1 was isolated as a pre-B cell interacting clone in the bone marrow stroma and named SIM [44]. Initially, STIM1 was thought to localize to the PM but later studies show that although a small percent localizes to the PM, STIM1 is primarily an ER protein and acts as a Ca^2+^ sensor that reports the state of filling of ER Ca^2+^ stores and underlies the first step in SOCE activation [41,45,46,47]. 

Structurally, STIM1 is an ER transmembrane protein with an ER luminal region encompassing a canonical EF-hand motif that senses ER Ca^2+^ concentration (Kd: 200–600 µM); and a sterile α-motif (SAM) that is essential for STIM dimerization [45,46,48,49,50]. The cytosolic portion of STIM1 is composed of three putative coiled-coils (CC1, CC2 and CC3), with CC2 and CC3 forming the domain that binds to and activates Orai1 (SOAR/CAD) [51,52,53,54,55]. 

Vertebrate genomes code for two STIM isoforms: STIM1 and STIM2, both of which are enriched in the ER membrane. Despite the high sequence conservation and the structural homology between STIM1 and STIM2, they differentially sense ER Ca^2+^ store levels with STIM2 sensing mild store depletion to maintain basal Ca^2+^ homeostasis, whereas STIM1 senses significant store depletion to activate SOCE [56,57,58]. This is mediated by a higher affinity of the EF-hand motif of STIM1 compared to STIM2 [58,59]. Furthermore, STIM1 aggregates with faster kinetics and interacts with Orai proteins more efficaciously than STIM2, resulting in more robust Ca^2+^ entry [60,61]. Hence, STIM2 is thought to play a housekeeping role contributing to maintaining ER Ca^2+^ concentration upon minimal to moderate store depletion, while STIM1 is considered the major ER Ca^2+^ sensor for strong store depletion [58,62]. Overexpression of STIM2 results in inhibition of SOCE [57] and interestingly loss of only STIM2 in lymphocytes leads to decreased cytokine production, arguing for an important role for the basal Ca^2+^ influx mediated by STIM2 in NFAT activation [63]. These effects appear to be mediated by two STIM2 splice variants that alternatively splice exon 9, resulting in differential effects on SOCE. STIM2.1 or STIM2β (754 aa) has an additional sequence inserted in its SOAR/CAD domain and does not by itself bind Orai1 and, as such, functions as a negative regulator of SOCE. In contrast, STIM2.2 or STIM2α (746 aa) functions as a positive modulator of SOCE and when overexpressed leads to constitutive activation of SOCE [64,65]. 

STIM1 which exists as a dimer at rest adopts an open activated confirmation after store depletion that exposes the SOAR/CAD domain, which binds to the Orai1 channel and gates it open (Figure 2). Orai1 is a highly Ca^2+^ selective channel at the PM with 4 transmembrane domains and cytosolic N- and C-termini [66]. Vertebrate genomes express 3 ORAI isoforms Orai1, Orai2 and Orai3, with Orai1 being the best characterized [67,68,69]. The ORAI channel is a hexamer with the pore lined by six TM1 domains [70]. Typically, SOCE activity is mediated by the interaction of STIM1 and Orai1; however, some studies reported STIM1 interaction with other partners including TRP channels and Orai1 with the secretory pathway Ca^2+^ ATPase SPCA2 to elicit constitutive SOCE and enhance the carcinogenesis process in human breast cancer [71,72]. 

## 6. SOCE Regulates Cell Migration by Modulating Focal Adhesion Dynamics

There is significant interest in the literature in the role of SOCE in cancer progression and metastasis with implication on the role of STIM1 and Orai1 in cell migration. This was first assessed in the context of breast cancer where STIM1 and Orai1 were shown to be important for breast cancer cell migration and metastasis to the lung using xenograft mouse models [73]. This study also documented for the first time the involvement of focal adhesion turnover as a molecular mechanism by which SOCE modulates cell migration. Knockdown of either STIM1 or Orai1 was associated with decreased cell migration and increased focal adhesion size and intensity, which would be expected to slow down cell migration [73]. Consistently, overexpression of STIM1 and STIM2 in the less aggressive MCF-7 breast cancer cells enhances cell migration and invasiveness [74]. 

In the context of cell migration STIM1 knockdown was shown to accelerate sheet cell migration in human umbilical vein endothelial cells (HUVEC) and its overexpression results in decreased migration [24]. In contrast, in the weakly adherent H1299 metastatic lung cancer cells STIM1 knockdown slightly inhibited cell migration [24]. This is consistent with other reports that show a reduction in cell migration potential following STIM1 knockdown [75,76,77] (see Table 1 and Table 2 for a comprehensive list). Tsai et al. elegantly teased out these differential effects by studying the role of SOCE on focal adhesions (FA) using either low or high fibronectin concentration to modulate extracellular matrix adhesion strength. They showed that SOCE enhances FA and, thus, adhesion to the ECM at the leading edge of migrating cells. SOCE inhibition resulted in inhibition of migration when H1299 cells were plated on low fibronectin but enhancement of migration on high fibronectin. They conclude that when adhesion to the ECM is weak, SOCE enhances cell migration by increasing FA. In contrast, when adhesion to the EMC is strong SOCE slows down migration by strengthening FA [24]. Consistently, in MDA-MB-231 breast cancer cells knockdown of either STIM1 or Orai1 slows down cell migration, which is due to impairment in the focal adhesion turnover [73]. These results are consistent with the important role of focal adhesion turnover at both the leading and lagging ends of the cell to modulate cell migration [78]. 

The above studies show a role for SOCE in modulating FA turnover. This was recently extended in a study focused on the role of the Arf family of small GTPases in cell migration. The GTPase activity of Arfs is modulated by guanine nucleotide exchange factors (GEF) and GTPase activating proteins (GAP), which increase nucleotide exchange or GTP hydrolysis, respectively. One of these Arf GEFs is IQSec1, which contains a Ca^2+^-calmodulin binding IQ motif [98]. IQSec1 binds to Arf5 and modulates its activity [98]. Knockdown of either IQSec1 or Arf5 enhanced focal adhesions and inhibited migration of the aggressive breast cancer cell line MDA-MB-231. The modulation of FA by Arf5/IQSec1 was through the Oxysterol-binding protein (OSBP)-related proteins ORP3, which localizes to ER-plasma membrane junctions in a SOCE-dependent fashion. Therefore, the disassembly of FA at the rear of migrating cells requires SOCE, which activates ORP3 recruitment to ER-PM junctions resulting in IQSec1/Arf5 activation. 

These studies argue that the modulation of FA by SOCE is polarized and diagonally opposed at the front and rear of migrating cells, but functionally culminates in supporting cell migration. At the leading edge of migrating cells SOCE strengthens FA and enhances cell migration, whereas at the trailing end SOCE results in FA disassembly, which is also required for cell migration (Figure 1). Interestingly and despite the role of SOCE at the rear of migrating cells, STIM1 and Orai1 are enriched at the leading edge of moving cells [24,82,83]. This finding is puzzling especially given the Ca^2+^ gradient in migrating cells with low Ca^2+^ at the front and the role of SOCE in FA disassembly at the rear. It was proposed that the SOCE machinery at the front of migrating cells is important to replenish Ca^2+^ stores depleted following Ca^2+^ transients mediated by TRPM7 with the associated Ca^2+^-induced Ca^2+^ release through IP_3_ receptors [99]. This is an interesting hypothesis that would imply that SOCE is capable of refilling Ca^2+^ stores in the front of a migrating cell without inducing a broad cytoplasmic Ca^2+^ rise. This argues for the involvement of Ca^2+^ tunneling, which allows for Ca^2+^ flowing through SOCE channels to fuel IP_3_-dependent Ca^2+^ release without inducing a cytoplasmic Ca^2+^ rise [19,100,101,102]. Ca^2+^ tunneling is a tightly coupled Ca^2+^ signaling modality that allows for Ca^2+^ flowing through SOCE channels to be taken up into the ER through the activity of the sarcoplasmic endoplasmic reticulum Ca^2+^ ATPase (SERCA) and then released through open IP_3_ receptors [19] (Figure 2). Tunneling amplifies and extends the SOCE signal throughout the cell cortex. There is indeed direct evidence for this model from a study on migrating pancreatic acinar cells [103]. Both STIM1 and IP_3_ receptors localize to the leading edge of migrating acinar cells. Intriguingly, IP_3_ receptors surround individual focal adhesions and IP_3_-dependent Ca^2+^ release enhances FA size. Furthermore, inhibition of either SOCE or IP_3_-dependent Ca^2+^ release inhibits migration [103]. These findings support a model where Ca^2+^ flowing through Orai channels at the leading edge of migrating cells is tunneled through IP_3_ receptors to FA to strengthen nascent adhesion. 

Another explanation for refilling ER stores at the leading edge without affecting the Ca^2+^ gradient is if SOCE-dependent Ca^2+^ influx was limited to the SOCE microdomain through SERCA-dependent uptake into the ER (Figure 2). This would depend on the stoichiometry of SERCA versus SOCE at the site of Ca^2+^ entry at the front of the cell and would require enough SERCA pumps to localize to the SOCE microdomain to take up the Ca^2+^ flowing through Orai channels into the ER, thus preventing spillover out of the SOCE microdomain [19,85,88]. 

Furthermore, given that the tight coupling between SOCE and FA at the rear end of migrating cells results in FA disassembly, one may postulate that the SOCE-dependent enhancement of FA at the front of the cell is indirect through intermediate effectors. Therefore, much remains to be learned about the mechanisms controlling SOCE activity in a polarized fashion in migrating cells. However, it is clear that tight modulation of SOCE spatially is important to support cell migration. 

## 7. Additional Mechanisms Involving SOCE in Cell Migration 

Alteration of calpain activity and spectrin processes could be another mechanism by which STIM1-specific siRNA decreased cell migration [90]. In fact, cytosolic Ca^2+^ transients stimulate the Ca^2+^ regulated protease calpain, which increases the disassembly rates via cleaving talin at the focal adhesion sites along with other focal adhesion protein such as paxillin, vinculin and zyxin [104]. In cervical cancer cells, STIM1 knockdown inhibited cell migration and significantly inhibited EGF-induced calpain activation [90]. This was also associated with inhibition of the ability of EGF to induce the phosphorylation of protein-rich tyrosine kinase 2 beta (PTK2B or PYK2) and the focal adhesion kinase (FAK), which are important regulators of focal adhesion dynamic [86,90]. 

Finally, a role for STIM1 phosphorylation has been proposed in regulating cell migration. Overexpression of a STIM1 mutant where the ERK1/2 phosphorylation sites (Ser575, Ser608 and Ser621) were mutated to alanines reduced cell migration [105]. Furthermore, phosphorylated STIM1 as detected by phospho-specific antibodies is enriched at the leading edge and membrane ruffles in migrating cells [86]. Both STIM1 and Orai1 have been proposed to interact with cortactin (CTTN), a major player in actin cytoskeleton remodeling [86,106]. Phosphorylation of STIM1 to spatially regulate SOCE would be an attractive regulatory approach as it is dynamic and can be readily controlled spatially. However, the recent generation of a non-phosphorylatable STIM1 mouse strain where all 10 Ser/Thr residues in the STIM1 C-terminal domain were replaced with Ala brings into question the importance of STIM1 phosphorylation in regulating cell migration [107]. If, indeed, STIM1 phosphorylation is critical for cell migration one would expect developmental pathologies in this mouse line as it would affect organ development. However, the mice are healthy with no overt phenotype and develop normally [107]. 

## 8. Disruption of Ca^2+^ Homeostasis in Cancer Cells

Interestingly, disruption of Ca^2+^ homeostasis has been repeatedly associated with cancer progression, which requires cellular migration to mediate metastasis. Ca^2+^ in the extracellular space is between 1–2 mM, in the cytosol ~100 nM and in the endoplasmic reticulum (ER) ranges between 100–800 µM [108]. Several cellular components cooperate to maintain Ca^2+^ homeostasis, including channels, transporters, receptors, downstream effectors and buffering proteins [109]. Disruptions of this homeostasis that lead to an elevation in basal Ca^2+^ levels or decrease ER Ca^2+^ have been associated with cancers and changes in the expression of specific Ca^2+^ pumps or channels have been documented in several tumor types [110]. This affects cell proliferation and migration, and decreases apoptosis, thus supporting tumor development [78,111,112,113]. 

Expression and function of members of the TRP channel superfamily, including TRPC1, TRPC6, TRPV1, TRPV2, TRPV6, TRPM7 and TRPM8 have been implicated in tumor growth and migration [22,26,30,114,115,116,117,118,119,120,121,122,123]. Similarly, several voltage-gated Ca^2+^ channels (T, L, N, P/Q and R-type VGCCs) have been associated with different cancer types such as melanoma, colon, prostate, and pancreatic cancers [124]. For instance, in the context of breast tumor, the T-type VGCC was found to be overexpressed in HER-2 positive SKBR cells that were resistant to trastuzumab and in luminal versus basal breast tumor in one study [125]. In another study, knockdown of the VGCC auxiliary subunit gamma 4 (CACNG4) in breast cancer cell lines reduced cell migration preferentially in the more aggressive MDA-MB-231 cells as compared to MCF7; and CACNG4 overexpression resulted in enhanced lung metastasis and death [126]. Furthermore, high expression levels of TRPM7 predict a poor outcome in breast cancers due to increased metastasis as confirmed using mouse xenograft model of human breast cancer [30,118]. 

In addition, to these Ca^2+^ signaling pathways store operated Ca^2+^ entry (SOCE) has also been implicated in cancer metastasis and tumor cell migration as will be discussed in further details below. This is consistent with the notion that Ca^2+^ influx plays an essential role in the tumorigenesis and metastasis [127,128]. 

## 9. SOCE Dependent Regulation of Cancer Cell Migration and Metastasis

Cancer metastasis is considered the end stage of the progression of any tumor [3,129,130,131]. It is composed of several steps that include infiltration of cancerous cells into the neighboring tissue, followed by intravasation as tumor cells undergo transendothelial migration through the vessel wall and, finally, extravasation and proliferation at the distant organ to form secondary tumors [130]. Despite cancer metastasis accounting for almost 90% of all cancer-related death, much remains to be learned regarding the molecular mechanisms underlying metastatic progression [3,131]. 

In addition to cytoskeletal remodeling, signaling cascades, ion channels and transporters have also been implicated in the metastatic cascade [132]. Such transport pathways modulate cell volume as well as Ca^2+^ and proton transport, which are important for cell migration. Among Ca^2+^ influx pathways, SOCE has been repeatedly implicated in the migration and proliferation of many cancer types such as cervical cancer, breast cancer and melanoma [75,76,117]. It has been proposed that this is due to constitutive activation of SOCE at low levels in cancerous cells given their lower ER Ca^2+^ content [133,134,135]. Furthermore, SOCE was found to modulate migration and invasion of various cancer cells, including colorectal, prostate, breast, esophageal, endometrial adenocarcinoma and glioma cells [75,90,121,122], as well as being involved in tumor proliferation, initiation and carcinogenesis [136,137]. 

As summarized in Table 1, knockdown of STIM1 or Orai1 in various cancer cells is associated with inhibition of cell migration, with some exceptions. Consistently overexpression enhances cell migration. This is inconsistent with the original identification of STIM1 as a candidate tumor suppressor as it maps to a region on Chromosome 11 that when deleted is associated with tumors [40,41]. Independently, through an elegant in vivo screen of a weak melanoma cell line in mice, Suyama et al. identifies STIM1 as a suppressor of tumor metastasis and showed that when STIM1 is knocked-down it resulted in faster cell migration in the wound healing assay [77]. This finding is supported by the lack of expression of STIM1 in rhabdomyosarcoma and rhabdoid cancer. In addition, overexpression of STIM1 in rhabdomyosarcoma and rhabdoid cancer cell lines results in cell death but has no effect in breast cancer cell lines [43], arguing for cell type specific effect of STIM1 expression in the context of cancer progression. Similarly, in prostate cancer cells Orai1 knockdown inhibited apoptosis and STIM1 or Orai1 expression enhances cell senescence [85,138], thus supporting a role for SOCE in cancer cell death. Consistently, STIM1 expression was substantially decreased in hyperplasia and tumor tissue at histological grade 3 and 4, compared to normal tissue [85]. In contrast, in prostate cancer cell lines (LNCaP, PC3 and DU145) STIM1 was expressed at higher levels compared with the hyperplasia cell line BPH-1 and this was associated with higher SOCE levels [85]. STIM1 expression level and SOCE activity were lower in the more malignant cell line PC3 compared to LNCaP and DU145 cells [85]. Furthermore, inhibition of SOCE reduced cell migration, invasiveness and/or proliferation in melanoma, lung, and breast cancer cells [75,117,129,130]. These findings argue that STIM1 and Orai1 play multiple roles in cancer cells and may have a different regulatory mechanism even in the same cancer type.

Clinically breast tumors with high expression of STIM1 and low expression of STIM2 were associated with poorer prognosis [69]. High levels of STIM1, STIM2 and SOCE were also documented in metastatic melanoma as compared to primary melanoma [76,80] and knockdown of Orai1 and/or STIM2 reduced melanoma cell migration and invasiveness [80]. However, another study showed lower levels of SOCE in invasive patient-derived melanomas compared to non-invasive melanoma and this downregulation appears to be due to PKC-dependent phosphorylation of Orai1 [139], arguing for a complex relationship between SOCE and melanoma progression. 

As is the case in cancer cells, modulation of the expression of STIM1/Orai1 has differential effects in other non-cancerous cell lines (Table 2). Knockdown of STIM1 or Orai1 alone or in combination, but not STIM2, in human bronchial smooth muscle cells leads to significant inhibition for PDGF-BB induced cell migration [97]. Similarly, knockdown of either STIM1 or Orai1 in HEK293 cells reduced cell migration to similar levels although their effects on FA adhesion turnover was different [92]. In vascular smooth muscle cells knockdown of STIM1 or Orai1 reduces proliferation and migration, but no effect was observed following knockdown of the other STIM/Orai isoforms [93,94]. However, in contrast to the mostly consistent finding of SOCE inhibition resulting in slower cell migration, surprisingly complete knockout of STIM1 using CRISPR/Cas9 genome editing in mouse embryonic fibroblast (MEF) cells was associated with faster cell migration in response to platelet-derived growth factor (PDGF) [95]. This is apparently due to enhanced STIM2 activation, which leads to increase Ca^2+^ influx. This finding argues that STIM2 upon complete loss of STIM1 can support Ca^2+^ influx to modulate cell migration [95].

Finally, knockdown of STIM1 in MEFs resulted in a significant reduction in the number of invadopodia/podosomes [96]. Invadopodia are dynamic F-actin rich membrane protrusions that are essential for cancer cell metastasis and invasion, while podosomes play an crucial role in the degradation of the extracellular matrix (ECM), thus, facilitating cell motility and invasion during metastasis [96]. SOCE was proposed to control Ca^2+^ levels at protrusion sites in migrating cells, thus, enhancing the reorganization of the cytoskeleton network and supporting both lamellipodia and filopodia formation [86]. Moreover, as podosomes recruit matrix metalloproteinases (MMPs) that help in the degradation process, decreasing the expression of STIM1 was found to significantly decrease the activity of MMP2 and MMP9, thus altering the invasion ability of the cells [96].

**Figure 3:**
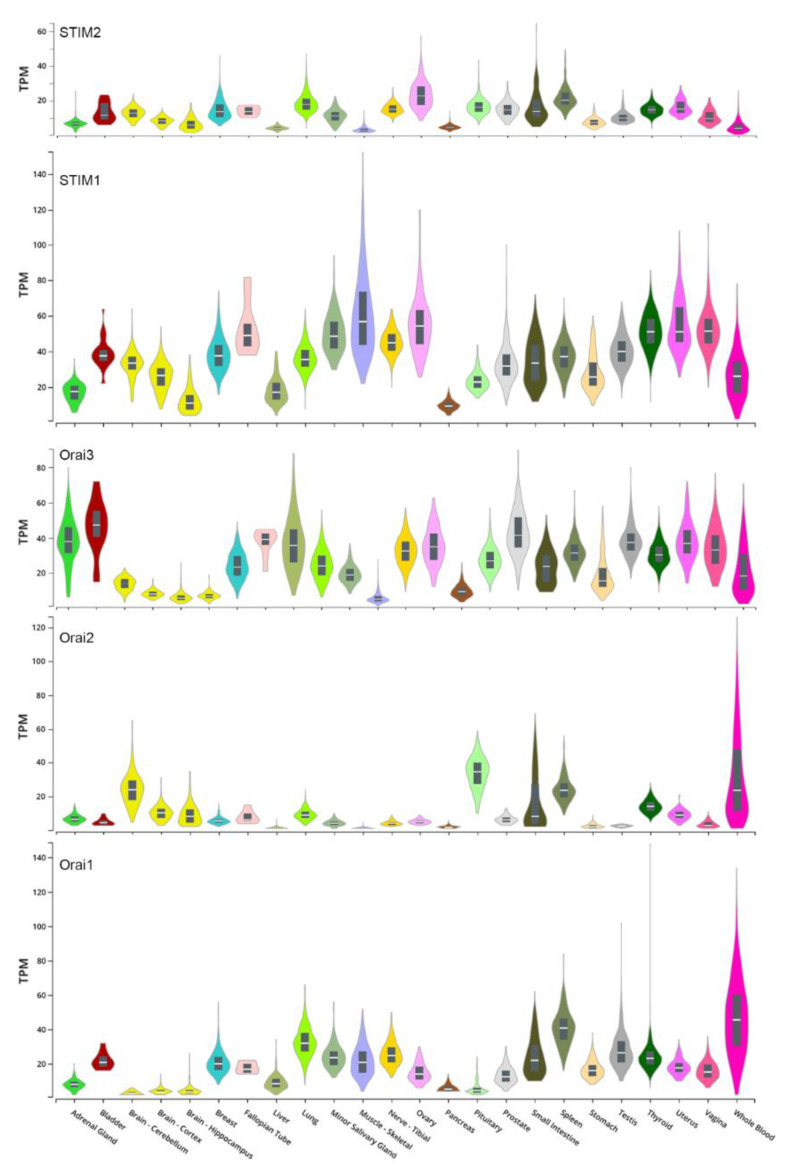
Expression levels of STIM and Orai isoforms in human organs. Data were obtained from the Genotype-Tissue Expression (GTEx) portal and are expressed as transcripts per million (TPM).

Collectively these studies show that the expression of STIM1 has differential effects in different cancers and sometimes as is the case for melanomas in the same cancer type. In most cases STIM1 downregulation inhibits metastasis, but there are clear examples that are difficult to ignore where STIM1 downregulation enhances metastasis and cell migration. In particular, in the example of the unbiased ribozyme screen using a weak melanoma cell line that identified STIM1 as a tumor suppressor [77]. Based on these divergent results one can conclude that modulating SOCE levels impacts cell migration and metastasis; however, the direction of this modulation appears to be cell and tissue type specific. Given the above discussion on the differential role of SOCE in the front and rear end of moving cells in regulating FA, it is tempting to speculate that SOCE modulation can differentially regulate FA and the actin cytoskeleton in a cell type specific fashion. Another issue to consider here that has not been carefully addressed in the cell migration literature is the established role of STIM1/Orai1 stoichiometry in modulating SOCE. SOCE levels have a nonlinear bell-shaped dependence on STIM1/Orai1 levels [140], where low or high STIM1/Orai1 ratios yield small SOCE. Furthermore, at high STIM1 expression levels Orai1 is trapped intracellularly and is no longer available to mediate SOCE at the PM [141]. This is because Orai1 recycles continuously between the PM and an intracellular vesicular pool with ~40% of the total Orai1 cellular pool localizing to the PM at steady state [141]. Therefore, depending on the initial stoichiometry of STIM1 and Orai1 in the cell or tissue of interest, knockdown of either protein could shift SOCE levels left or right along the stoichiometry bell curve, thus resulting in differential modulation of SOCE and by extension cell migration. Indeed, the absolute and relative expression levels of STIM and Orai isoforms to each other varies significantly among different tissues as shown in Figure 3 as an example in humans. Furthermore, we do not know whether the different knockdown approaches used experimentally result is homogenous downregulation of SOCE proteins, or whether they exhibit spatially different downregulation levels.

## 10. SOCE and the Epithelial to Mesenchymal Transition (EMT)

SOCE may also be involved in the epithelial to mesenchymal transition (EMT). In colorectal cancer cell lines STIM1 knockdown resulted in increased expression of E-cadherin and β-catenin and decrease in the level of vimentin and fibronectin [82]. β-catenin and E-cadherin are epithelial markers whereas fibronectin and vimentin are mesenchymal markers. STIM1 knock-down was also associated with decreased metastasis to the lung and its overexpression in increased metastasis [82]. Knockdown of STIM1 in prostate cancer cells was related to EMT suppression [84] and in breast and lung cancer cells the expression of E-cadherin, Snail and Vimentin were regulated by STIM1/2 expression [74,79]. Using the gastric cancer cell lines BGC-803 and MKN-45 cells, the decreased migration and invasion was associated with a decrease in the vimentin and fibronectin expression and an increased in E-cadherin expression upon knockdown of Orai1 and/or STIM1 [89].

STIM2 was also implicated in breast cancer metastasis through supporting EMT [83]. STIM2 knockdown inhibited breast cancer cell migration and metastasis in xenograft models [83]. This was associated with inhibition of nuclear factor of activated T cells 1 (NFAT1) and its downstream expression of EMT markers. In contrast, STIM2 overexpression enhanced metastasis and resulted in activation of NFAT1 and TGF-β signaling [83]. These results argue that STIM2, presumably through regulating basal cytosolic Ca^2+^ levels, activates NFAT1, which in turn induces TGF-β1 expression to promote EMT and enhance cell migration and metastasis of breast cancer cells. The STIM1 effect on cell migration was also attributed to NFATc1 activation in both osteosarcoma and gastric cancer cells [87,88]. Additionally, in human primary gastric tumors higher expression of Orai1 and STIM1 was associated with poorer prognosis [89]. The link between STIM1/STIM2 expression levels and NFAT is important in potentially explaining the differential effects observed following changes in expression of these proteins on cell migration. This is because different cell types have distinct transcriptional programs that can be altered based on NFAT modulation.

## 11. Concluding Remarks

Ca^2+^ signaling is intimately connected to the regulation of cell migration and regulates both adhesion to the ECM as well as cytoskeletal remodeling. The Ca^2+^ signaling machinery is polarized in migrating cells and this polarization has functional consequences on migration. In particular, SOCE has differential effects at the front and rear of migrating cells in terms of regulation of focal adhesion dynamics. Furthermore, modulation of either STIM1, STIM2 or Orai1 expression may be associated with remodeling of the cell’s transcription program given the well-documented induction of NFAT through calcineurin activation downstream of SOCE [142]. This may be important in explaining the differential results obtained in different cell lines and tumors.

The effect of modulating SOCE in terms of cell migration and tumor metastasis is tissue and cell type dependent with sometimes opposing effects. For example, STIM1 was originally isolated as a tumor suppressor and was validated as such in an independent screen [42,43,125], yet in the context of several cancers including breast cancer STIM1 and Orai1 are crucial for metastasis [73]. This argues for a potential role for STIM1 that is independent of mediating Ca^2+^ entry through SOCE. Furthermore, SOCE does not function independently in modulating Ca^2+^ signaling dynamics in migrating cells. Hence, changes in the expression levels of SOCE components may be countered differentially in terms of modulating the activity/expression of other Ca^2+^ signaling components involved in cell migration (TPRM7, VGCC, PMCA, IP_3_ receptor, etc.). In turn these Ca^2+^ channels/transporters would affect Ca^2+^ dynamics and accordingly differentially modulate the actin cytoskeleton and focal adhesions with functional consequences on cell migration. Therefore, a comprehensive understanding of the role of SOCE in cell migration will require more in-depth mechanistic studies on multiple cell types to better define not only the molecular regulation of SOCE in the polarized migrating cell, but also to define the SOCE downstream effectors in the context of cell migration.

## Figures and Tables

**Table 1:** Effect of modulation of the expression of STIM and Orai isoforms on the migration of different cancer cells.

Cancerous Cells	Cell Lines	Perturbation	Result	Ref.
Non-small-cell lung cancer (NSCLC)	A549 SK-MES-1	STIM1 knockdown	Reduced proliferation	[75]
	A549	STIM1 knockdown	Reduced migration & metastasis	[79]
	H1299	STIM1 knockdown	Slightly decreased migration	[24]
Melanoma	SK-Mel-2 SK-Mel-24	STIM1/Orai1 knockdown	Reduced migration & metastasis	[76]
	B16F0 cells	STIM1 knockdown	Enhanced migration	[77]
	non-commercial WM3734 melanoma cell lines non-commercial WM3734 melanoma cell lines non-commercial WM3734 melanoma cell lines WM3734 non-commercial	STIM2/Orai1 knockdown	Reduced migration & invasiveness	[80]
Colorectal Cancer	Primary liver metastasis	STIM1/Orai1/Orai3 knockdown	No effect on migration	[81]
	SW620	STIM1 knockdown	Reduced migration & invasiveness	[82]
	SW480	STIM1 overexpression	Enhanced migration & invasiveness	
Breast Cancer	MDA-MB-231	STIM1/Orai1 knockdown	Reduced migration	[73]
	MDA-MB-231	STIM2 knockdown	Reduced migration	[83]
	MCF-7	STIM1/STIM2 overexpression	Enhanced migration & invasiveness	[74]
Prostate Cancer	PC-3 DU-145	STIM1 knockdown	Reduced migration and invasion	[84]
	DU145 PC3	STIM1/Orai1 overexpression	Enhance migration & cell growth	[85]
Osteosarcoma	U2OS	STIM1/Orai1 knockdown	Reduced migration	[86]
	143B U2OS	STIM1 knockout	Reduced migration	[87]
Gastric Cancer	MKN-45 SGC-7901	STIM1knockdown	Reduce migration & invasiveness	[88]
	MKN-45 BGC-803	STIM1/Orai1 knockdown	Reduce migration & invasiveness	[89]
Cervical Cancer	SiHa CaSki	STIM1 knockdown and overexpression	KD reduced migration Overexpression increased migration & invasion	[90]
Human oesophageal cancer	(KYSE-30)	Orai1 knockdown	Reduced migration	[91]

**Table 2:** Effect of modulation of the expression of STIM and Orai isoforms on the migration of different cell lines.

Cell Lines	Method	Result	Ref.
Human embryonic kidney cells (HEK293)	STIM1/Orai1 knockdown	Reduced migration	[92]
Vascular smooth muscle cells (VSMCs)	STIM1/STIM2/Orai1/Orai2/Orai3 knockdown	STIM1/Orai1 reduced migration No effect for STIM2/Orai2/Orai3	[93,94]
Mouse embryonic fibroblasts (MEF)	STIM1 knockout	Enhanced migration	[95]
	STIM1 knockdown	Reduced invasion	[96]
Human umbilical vein endothelial cells (HUVEC)	STIM1 knockdown	Enhanced migration	[24]
	STIM1 overexpression	Decreased migration	
Primary human bronchial smooth muscle cells	STIM1/STIM2/Orai1 knockdown	STIM1/Orai1 reduced migration STIM2 no effect	[97]

## Data Availability

All data presented in this review are present within the article or associated references.

## Abbreviations

CBP	Calcium Binding Proteins
PMCA	Plasma Membrane Ca^2+^ ATPase
MLCK	Myosin Light Chain Kinase
PKA	Protein Kinase A
ER	Endoplasmic Reticulum
SR	Sarcoplasmic Reticulum
SOCE	Store-Operated Calcium Entry
STIM1	Stromal Interation Molecule 1
PM	Plasma Membrane
CRAC	Ca^2+^ Release-Activated Ca^2+^ channels
SOAR	STIM1-Orai1-Activating Region
SAM	Sterile α-Motif
GpcR	G-Protein Coupled Receptors
PLC	Phospholipase C
PIP2	Phosphatidylinositol 4,5-Bisphosphate
IP3	Inositol 1,4,5-Trisphosphate
PKC	Protein Kinase C
ORAI	Calcium Release-Activated Calcium Channel Protein
EB1	End-Binding Protein 1
MT	Microtubule
HBC	Human Breast Cancer
ECM	Extracellular Matrix
ERM	Ezrin, Radixin and Moesin
MPA	Membrane-Proximal F-Actin
TRPM7	Transient Receptor Potential Melastatin Channel 7
CaM	Calmodulin
CACNG4	Calcium Voltage-Gated Channel Auxillary Subunit Gamma 4
pCRC	Primary Colorectal Tumor Cells
MEF	Mouse Embryonic Fibroblast
PDGF	Platelet-Derived Growth Factor
PTK2B	Protein-Rich Tyrosine Kinase 2 Beta
FAK	Focal Adhesion Kinase
PDGF	Platelet-Derived Growth Factor
HUVEC	Human Umbilical Vein Endothelial Cells
EMT	Epithelial-to Mesenchymal Transition
TGF-β	Transforming Growth Factor-β
MMP	Matrix Metalloproteinases

## References

[1] Shellard A., Mayor R. (2020). All Roads Lead to Directional Cell Migration. Trends Cell Biol..

[2] Kraljevic Pavelic S., Sedic M., Bosnjak H., Spaventi S., Pavelic K. (2011). Metastasis: New perspectives on an old problem. Mol. Cancer.

[3] Mo P., Yang S. (2018). The store-operated calcium channels in cancer metastasis: From cell migration, invasion to metastatic colonization. Front. Biosci. (Landmark Ed.).

[4] Huang H.K., Lin Y.H., Chang H.A., Lai Y.S., Chen Y.C., Huang S.C., Chou C.Y., Chiu W.T. (2020). Chemoresistant ovarian cancer enhances its migration abilities by increasing store-operated Ca^2+^ entry-mediated turnover of focal adhesions. J. Biomed. Sci..

[5] Chang S.J., Chen Y.C., Yang C.H., Huang S.C., Huang H.K., Li C.C., Harn H.I., Chiu W.T. (2017). Revealing the three dimensional architecture of focal adhesion components to explain Ca^2+^-mediated turnover of focal adhesions. Biochim. Biophys. Acta Gen Subj..

[6] Derouiche S., Warnier M., Mariot P., Gosset P., Mauroy B., Bonnal J.L., Slomianny C., Delcourt P., Prevarskaya N., Roudbaraki M. (2013). Bisphenol A stimulates human prostate cancer cell migration via remodelling of calcium signalling. Springerplus.

[7] Ridley A.J., Schwartz M.A., Burridge K., Firtel R.A., Ginsberg M.H., Borisy G., Parsons J.T., Horwitz A.R. (2003). Cell migration: Integrating signals from front to back. Science.

[8] Ridley A.J. (2011). Life at the leading edge. Cell.

[9] Yang S., Huang X.Y. (2005). Ca^2+^ influx through L-type Ca^2+^ channels controls the trailing tail contraction in growth factor-induced fibroblast cell migration. J. Biol. Chem..

[10] Mayor R., Etienne-Manneville S. (2016). The front and rear of collective cell migration. Nat. Rev. Mol. Cell Biol..

[11] Petrie R.J., Doyle A.D., Yamada K.M. (2009). Random versus directionally persistent cell migration. Nat. Rev. Mol. Cell Biol..

[12] Fehon R.G., McClatchey A.I., Bretscher A. (2010). Organizing the cell cortex: The role of ERM proteins. Nat. Rev. Mol. Cell Biol..

[13] Diz-Muñoz A., Krieg M., Bergert M., Ibarlucea-Benitez I., Muller D.J., Paluch E., Heisenberg C.P. (2010). Control of directed cell migration in vivo by membrane-to-cortex attachment. PLoS Biol..

[14] Liu Y.J., Le Berre M., Lautenschlaeger F., Maiuri P., Callan-Jones A., Heuzé M., Takaki T., Voituriez R., Piel M. (2015). Confinement and low adhesion induce fast amoeboid migration of slow mesenchymal cells. Cell.

[15] Bravo-Cordero J.J., Magalhaes M.A., Eddy R.J., Hodgson L., Condeelis J. (2013). Functions of cofilin in cell locomotion and invasion. Nat. Rev. Mol. Cell Biol..

[16] Bisaria A., Hayer A., Garbett D., Cohen D., Meyer T. (2020). Membrane-proximal F-actin restricts local membrane protrusions and directs cell migration. Science.

[17] Oser M., Condeelis J. (2009). The cofilin activity cycle in lamellipodia and invadopodia. J. Cell Biochem..

[18] Berridge M.J. (2002). The endoplasmic reticulum: A multifunctional signaling organelle. Cell Calcium.

[19] Courjaret R.J., Machaca K. (2020). Expanding the Store-operated Ca^2+^ Entry microdomain through Ca^2+^ tunneling. Current Opinion in Physiology.

[20] Venkatachalam K., Montell C. (2007). TRP channels. Annu. Rev. Biochem..

[21] Prakriya M., Lewis R.S. (2015). Store-Operated Calcium Channels. Physiol. Rev..

[22] Brundage R.A., Fogarty K.E., Tuft R.A., Fay F.S. (1991). Calcium Gradients Underlying Polarization and Chemotaxis of Eosinophils. Science.

[23] Gilbert S.H., Perry K., Fay F.S. (1994). Mediation of chemoattractant-induced changes in [Ca^2+^]i and cell shape, polarity, and locomotion by InsP3, DAG, and protein kinase C in newt eosinophils. J. Cell Biol..

[24] Tsai F.C., Seki A., Yang H.W., Hayer A., Carrasco S., Malmersjo S., Meyer T. (2014). A polarized Ca^2+^, diacylglycerol and STIM1 signalling system regulates directed cell migration. Nat. Cell Biol..

[25] Kim J.M., Lee M., Kim N., Heo W.D. (2016). Optogenetic toolkit reveals the role of Ca^2+^ sparklets in coordinated cell migration. Proc. Natl. Acad. Sci. USA.

[26] Wei C., Wang X., Chen M., Ouyang K., Song L.S., Cheng H. (2009). Calcium flickers steer cell migration. Nature.

[27] Visser D., Langeslag M., Kedziora K.M., Klarenbeek J., Kamermans A., Horgen F.D., Fleig A., van Leeuwen F.N., Jalink K. (2013). TRPM7 triggers Ca^2+^ sparks and invadosome formation in neuroblastoma cells. Cell Calcium..

[28] Giannone G., Ronde P., Gaire M., Haiech J., Takeda K. (2002). Calcium oscillations trigger focal adhesion disassembly in human U87 astrocytoma cells. J. Biol. Chem..

[29] Tsai F.C., Meyer T. (2012). Ca^2+^ pulses control local cycles of lamellipodia retraction and adhesion along the front of migrating cells. Curr. Biol..

[30] Middelbeek J., Kuipers A.J., Henneman L., Visser D., Eidhof I., van Horssen R., Wieringa B., Canisius S.V., Zwart W., Wessels L.F. (2012). TRPM7 is required for breast tumor cell metastasis. Cancer Res..

[31] Minton K. (2014). Cell migration: Coordinating calcium signalling. Nat. Rev. Mol. Cell Biol..

[32] Pollard T.D., Borisy G.G. (2003). Cellular motility driven by assembly and disassembly of actin filaments. Cell.

[33] Evans J.H., Falke J.J. (2007). Ca^2+^ influx is an essential component of the positive-feedback loop that maintains leading-edge structure and activity in macrophages. Proc. Natl. Acad. Sci. USA.

[34] Price L.S., Langeslag M., ten Klooster J.P., Hordijk P.L., Jalink K., Collard J.G. (2003). Calcium signaling regulates translocation and activation of Rac. J. Biol. Chem..

[35] Kholmanskikh S.S., Koeller H.B., Wynshaw-Boris A., Gomez T., Letourneau P.C., Ross M.E. (2006). Calcium-dependent interaction of Lis1 with IQGAP1 and Cdc42 promotes neuronal motility. Nat. Neurosci..

[36] Ohta Y., Nishida E., Sakai H. (1986). Type II Ca^2+^/calmodulin-dependent protein kinase binds to actin filaments in a calmodulin-sensitive manner. FEBS Lett..

[37] Larsson C. (2006). Protein kinase C and the regulation of the actin cytoskeleton. Cell Signal..

[38] Hoffman L., Farley M.M., Waxham M.N. (2013). Calcium-calmodulin-dependent protein kinase II isoforms differentially impact the dynamics and structure of the actin cytoskeleton. Biochemistry.

[39] Sotelo-Avila C., Gooch W.M. (1976). Neoplasms associated with the Beckwith-Wiedemann syndrome. Perspect Pediatr. Pathol..

[40] Parker N.J., Begley C.G., Smith P.J., Fox R.M. (1996). Molecular cloning of a novel human gene (D11S4896E) at chromosomal region 11p15.5. Genomics.

[41] Manji S.S., Parker N.J., Williams R.T., van Stekelenburg L., Pearson R.B., Dziadek M., Smith P.J. (2000). STIM1: A novel phosphoprotein located at the cell surface. Biochim. Biophys. Acta.

[42] Hu R.J., Lee M.P., Connors T.D., Johnson L.A., Burn T.C., Su K., Landes G.M., Feinberg A.P. (1997). A 2.5-Mb transcript map of a tumor-suppressing subchromosomal transferable fragment from 11p15.5, and isolation and sequence analysis of three novel genes. Genomics.

[43] Sabbioni S., Barbanti-Brodano G., Croce C.M., Negrini M. (1997). GOK: A gene at 11p15 involved in rhabdomyosarcoma and rhabdoid tumor development. Cancer Res..

[44] Oritani K., Kincade P.W. (1996). Identification of stromal cell products that interact with pre-B cells. J. Cell Biol..

[45] Liou J., Kim M.L., Heo W.D., Jones J.T., Myers J.W., Ferrell J.E., Meyer T. (2005). STIM is a Ca^2+^ sensor essential for Ca^2+^-store-depletion-triggered Ca^2+^ influx. Curr. Biol..

[46] Zhang S.L., Yu Y., Roos J., Kozak J.A., Deerinck T.J., Ellisman M.H., Stauderman K.A., Cahalan M.D. (2005). STIM1 is a Ca^2+^ sensor that activates CRAC channels and migrates from the Ca^2+^ store to the plasma membrane. Nature.

[47] Soboloff J., Spassova M.A., Tang X.D., Hewavitharana T., Xu W., Gill D.L. (2006). Orai1 and STIM reconstitute store-operated calcium channel function. J. Biol. Chem..

[48] Roos J., DiGregorio P.J., Yeromin A.V., Ohlsen K., Lioudyno M., Zhang S., Safrina O., Kozak J.A., Wagner S.L., Cahalan M.D. (2005). STIM1, an essential and conserved component of store-operated Ca^2+^ channel function. J. Cell Biol..

[49] Lopez E., Frischauf I., Jardin I., Derler I., Muik M., Cantonero C., Salido G.M., Smani T., Rosado J.A., Redondo P.C. (2019). STIM1 phosphorylation at Y(316) modulates its interaction with SARAF and the activation of SOCE and I (CRAC). J. Cell Sci..

[50] Stathopulos P.B., Li G.Y., Plevin M.J., Ames J.B., Ikura M. (2006). Stored Ca^2+^ depletion-induced oligomerization of stromal interaction molecule 1 (STIM1) via the EF-SAM region: An initiation mechanism for capacitive Ca^2+^ entry. J. Biol. Chem..

[51] Fahrner M., Muik M., Schindl R., Butorac C., Stathopulos P., Zheng L., Jardin I., Ikura M., Romanin C. (2014). A coiled-coil clamp controls both conformation and clustering of stromal interaction molecule 1 (STIM1). J. Biol. Chem..

[52] Korzeniowski M.K., Manjarres I.M., Varnai P., Balla T. (2010). Activation of STIM1-Orai1 involves an intramolecular switching mechanism. Sci. Signal.

[53] Zhou Y., Srinivasan P., Razavi S., Seymour S., Meraner P., Gudlur A., Stathopulos P.B., Ikura M., Rao A., Hogan P.G. (2013). Initial activation of STIM1, the regulator of store-operated calcium entry. Nat. Struct. Mol. Biol..

[54] Park C.Y., Hoover P.J., Mullins F.M., Bachhawat P., Covington E.D., Raunser S., Walz T., Garcia K.C., Dolmetsch R.E., Lewis R.S. (2009). STIM1 clusters and activates CRAC channels via direct binding of a cytosolic domain to Orai1. Cell.

[55] Yuan J.P., Zeng W., Dorwart M.R., Choi Y.J., Worley P.F., Muallem S. (2009). SOAR and the polybasic STIM1 domains gate and regulate Orai channels. Nat. Cell Biol..

[56] Chen Y.F., Chen L.H., Shen M.R. (2019). The distinct role of STIM1 and STIM2 in the regulation of store-operated Ca^2+^ entry and cellular function. J. Cell Physiol..

[57] Parvez S., Beck A., Peinelt C., Soboloff J., Lis A., Monteilh-Zoller M., Gill D.L., Fleig A., Penner R. (2008). STIM2 protein mediates distinct store-dependent and store-independent modes of CRAC channel activation. FASEB J..

[58] Brandman O., Liou J., Park W.S., Meyer T. (2007). STIM2 is a feedback regulator that stabilizes basal cytosolic and endoplasmic reticulum Ca^2+^ levels. Cell.

[59] Zheng L., Stathopulos P., Li G.Y., Ikura M. (2008). Biophysical characterization of the EF-hand and SAM domain containing Ca^2+^ sensory region of STIM1 and STIM2. Biochem. Biophys. Res. Commun..

[60] Stathopulos P.B., Zheng L., Ikura M. (2009). Stromal interaction molecule (STIM) 1 and STIM2 calcium sensing regions exhibit distinct unfolding and oligomerization kinetics. J. Biol. Chem..

[61] Wang X., Wang Y., Zhou Y., Hendron E., Mancarella S., Andrake M.D., Rothberg B.S., Soboloff J., Gill D.L. (2014). Distinct Orai-coupling domains in STIM1 and STIM2 define the Orai-activating site. Nat. Commun..

[62] Bird G.S., Hwang S.Y., Smyth J.T., Fukushima M., Boyles R.R., Putney J.W. (2009). STIM1 is a calcium sensor specialized for digital signaling. Curr. Biol..

[63] Oh-Hora M., Yamashita M., Hogan P.G., Sharma S., Lamperti E., Chung W., Prakriya M., Feske S., Rao A. (2008). Dual functions for the endoplasmic reticulum calcium sensors STIM1 and STIM2 in T cell activation and tolerance. Nat. Immunol..

[64] Miederer A.M., Alansary D., Schwär G., Lee P.H., Jung M., Helms V., Niemeyer B.A. (2015). A STIM2 splice variant negatively regulates store-operated calcium entry. Nat. Commun..

[65] Rana A., Yen M., Sadaghiani A.M., Malmersjö S., Park C.Y., Dolmetsch R.E., Lewis R.S. (2015). Alternative splicing converts STIM2 from an activator to an inhibitor of store-operated calcium channels. J. Cell Biol..

[66] Prakriya M. (2013). Store-operated Orai channels: Structure and function. Curr. Top Membr..

[67] Feske S., Gwack Y., Prakriya M., Srikanth S., Puppel S.H., Tanasa B., Hogan P.G., Lewis R.S., Daly M., Rao A. (2006). A mutation in Orai1 causes immune deficiency by abrogating CRAC channel function. Nature.

[68] Prakriya M., Feske S., Gwack Y., Srikanth S., Rao A., Hogan P.G. (2006). Orai1 is an essential pore subunit of the CRAC channel. Nature.

[69] McAndrew D., Grice D.M., Peters A.A., Davis F.M., Stewart T., Rice M., Smart C.E., Brown M.A., Kenny P.A., Roberts-Thomson S.J. (2011). ORAI1-mediated calcium influx in lactation and in breast cancer. Mol. Cancer Ther..

[70] Hou X., Pedi L., Diver M.M., Long S.B. (2012). Crystal structure of the calcium release-activated calcium channel Orai. Science.

[71] Feng M., Grice D.M., Faddy H.M., Nguyen N., Leitch S., Wang Y., Muend S., Kenny P.A., Sukumar S., Roberts-Thomson S.J. (2010). Store-independent activation of Orai1 by SPCA2 in mammary tumors. Cell.

[72] Zeng W., Yuan J.P., Kim M.S., Choi Y.J., Huang G.N., Worley P.F., Muallem S. (2008). STIM1 gates TRPC channels, but not Orai1, by electrostatic interaction. Mol. Cell.

[73] Yang S., Zhang J.J., Huang X.Y. (2009). Orai1 and STIM1 are critical for breast tumor cell migration and metastasis. Cancer Cell.

[74] Zhang S., Miao Y., Zheng X., Gong Y., Zhang J., Zou F., Cai C. (2017). STIM1 and STIM2 differently regulate endogenous Ca^2+^ entry and promote TGF-β-induced EMT in breast cancer cells. Biochem. Biophys. Res. Commun..

[75] Yang Y., Jiang Z., Wang B., Chang L., Liu J., Zhang L., Gu L. (2017). Expression of STIM1 is associated with tumor aggressiveness and poor prognosis in breast cancer. Pathol. Res. Pract..

[76] Umemura M., Baljinnyam E., Feske S., De Lorenzo M.S., Xie L.H., Feng X., Oda K., Makino A., Fujita T., Yokoyama U. (2014). Store-operated Ca^2+^ entry (SOCE) regulates melanoma proliferation and cell migration. PLoS ONE.

[77] Suyama E., Wadhwa R., Kaur K., Miyagishi M., Kaul S.C., Kawasaki H., Taira K. (2004). Identification of metastasis-related genes in a mouse model using a library of randomized ribozymes. J. Biol. Chem..

[78] Prevarskaya N., Skryma R., Shuba Y. (2011). Calcium in tumour metastasis: New roles for known actors. Nat. Rev.Cancer.

[79] Wang Y., Wang H., Pan T., Li L., Li J., Yang H. (2017). STIM1 silencing inhibits the migration and invasion of A549 cells. Mol. Med. Rep..

[80] Stanisz H., Saul S., Muller C.S., Kappl R., Niemeyer B.A., Vogt T., Hoth M., Roesch A., Bogeski I. (2014). Inverse regulation of melanoma growth and migration by Orai1/STIM2-dependent calcium entry. Pigment. Cell Melanoma Res..

[81] Zuccolo E., Laforenza U., Ferulli F., Pellavio G., Scarpellino G., Tanzi M., Turin I., Faris P., Lucariello A., Maestri M. (2018). Stim and Orai mediate constitutive Ca^2+^ entry and control endoplasmic reticulum Ca^2+^ refilling in primary cultures of colorectal carcinoma cells. Oncotarget.

[82] Zhang Z., Liu X., Feng B., Liu N., Wu Q., Han Y., Nie Y., Wu K., Shi Y., Fan D. (2015). STIM1, a direct target of microRNA-185, promotes tumor metastasis and is associated with poor prognosis in colorectal cancer. Oncogene.

[83] Miao Y., Shen Q., Zhang S., Huang H., Meng X., Zheng X., Yao Z., He Z., Lu S., Cai C. (2019). Calcium-sensing stromal interaction molecule 2 upregulates nuclear factor of activated T cells 1 and transforming growth factor-β signaling to promote breast cancer metastasis. Breast Cancer Res..

[84] Zhou Y., Gu P., Li J., Li F., Zhu J., Gao P., Zang Y., Wang Y., Shan Y., Yang D. (2017). Suppression of STIM1 inhibits the migration and invasion of human prostate cancer cells and is associated with PI3K/Akt signaling inactivation. Oncol. Rep..

[85] Xu Y., Zhang S., Niu H., Ye Y., Hu F., Chen S., Li X., Luo X., Jiang S., Liu Y. (2015). STIM1 accelerates cell senescence in a remodeled microenvironment but enhances the epithelial-to-mesenchymal transition in prostate cancer. Sci. Rep..

[86] Lopez-Guerrero A.M., Tomas-Martin P., Pascual-Caro C., Macartney T., Rojas-Fernandez A., Ball G., Alessi D.R., Pozo-Guisado E., Martin-Romero F.J. (2017). Regulation of membrane ruffling by polarized STIM1 and ORAI1 in cortactin-rich domains. Sci. Rep..

[87] Zang J., Zuo D., Shogren K.L., Gustafson C.T., Zhou Z., Thompson M.A., Guo R., Prakash Y.S., Lu L., Guo W. (2019). STIM1 expression is associated with osteosarcoma cell survival. Chin. J. Cancer Res..

[88] Xu J.M., Zhou Y., Gao L., Zhou S.X., Liu W.H., Li X.A. (2016). Stromal interaction molecule 1 plays an important role in gastric cancer progression. Oncol. Rep..

[89] Xia J., Wang H., Huang H., Sun L., Dong S., Huang N., Shi M., Bin J., Liao Y., Liao W. (2016). Elevated Orai1 and STIM1 expressions upregulate MACC1 expression to promote tumor cell proliferation, metabolism, migration, and invasion in human gastric cancer. Cancer Lett..

[90] Chen Y.F., Chiu W.T., Chen Y.T., Lin P.Y., Huang H.J., Chou C.Y., Chang H.C., Tang M.J., Shen M.R. (2011). Calcium store sensor stromal-interaction molecule 1-dependent signaling plays an important role in cervical cancer growth, migration, and angiogenesis. Proc. Natl. Acad. Sci. USA.

[91] Zhu H., Zhang H., Jin F., Fang M., Huang M., Yang C.S., Chen T., Fu L., Pan Z. (2014). Elevated Orai1 expression mediates tumor-promoting intracellular Ca^2+^ oscillations in human esophageal squamous cell carcinoma. Oncotarget.

[92] Schäfer C., Rymarczyk G., Ding L., Kirber M.T., Bolotina V.M. (2012). Role of molecular determinants of store-operated Ca^2+^ entry (Orai1, phospholipase A2 group 6, and STIM1) in focal adhesion formation and cell migration. J. Biol. Chem..

[93] Potier M., Gonzalez J.C., Motiani R.K., Abdullaev I.F., Bisaillon J.M., Singer H.A., Trebak M. (2009). Evidence for STIM1- and Orai1-dependent store-operated calcium influx through ICRAC in vascular smooth muscle cells: Role in proliferation and migration. FASEB J..

[94] Bisaillon J.M., Motiani R.K., Gonzalez-Cobos J.C., Potier M., Halligan K.E., Alzawahra W.F., Barroso M., Singer H.A., Jourd’heuil D., Trebak M. (2010). Essential role for STIM1/Orai1-mediated calcium influx in PDGF-induced smooth muscle migration. Am. J. Physiol. Cell Physiol..

[95] Huang T.Y., Lin Y.H., Chang H.A., Yeh T.Y., Chang Y.H., Chen Y.F., Chen Y.C., Li C.C., Chiu W.T. (2018). STIM1 Knockout Enhances PDGF-Mediated Ca^2+^ Signaling through Upregulation of the PDGFR(-)PLCgamma(-)STIM2 Cascade. Int. J. Mol. Sci..

[96] Chen Y.W., Lai C.S., Chen Y.F., Chiu W.T., Chen H.C., Shen M.R. (2017). STIM1-dependent Ca^2+^ signaling regulates podosome formation to facilitate cancer cell invasion. Sci. Rep..

[97] Suganuma N., Ito S., Aso H., Kondo M., Sato M., Sokabe M., Hasegawa Y. (2012). STIM1 regulates platelet-derived growth factor-induced migration and Ca^2+^ influx in human airway smooth muscle cells. PLoS ONE.

[98] D’Souza R.S., Lim J.Y., Turgut A., Servage K., Zhang J., Orth K., Sosale N., Lazzara M., Allegood J., Casanova J.E. (2020). Calcium-stimulated disassembly of focal adhesions mediated by an ORP3/IQSec1 complex. Elife.

[99] Tsai F.C., Kuo G.H., Chang S.W., Tsai P.J. (2015). Ca^2+^ signaling in cytoskeletal reorganization, cell migration, and cancer metastasis. Biomed Res. Int..

[100] Courjaret R., Dib M., Machaca K. (2018). Spatially restricted subcellular Ca^2+^ signaling downstream of store-operated calcium entry encoded by a cortical tunneling mechanism. Sci. Rep..

[101] Petersen O.H., Courjaret R., Machaca K. (2017). Ca^2+^ tunnelling through the ER lumen as a mechanism for delivering Ca^2+^ entering via store-operated Ca^2+^ channels to specific target sites. J. Physiol..

[102] Taylor C.W., Machaca K. (2019). IP3 receptors and store-operated Ca^2+^ entry: A license to fill. Curr. Opin. Cell Biol..

[103] Okeke E., Parker T., Dingsdale H., Concannon M., Awais M., Voronina S., Molgo J., Begg M., Metcalf D., Knight A.E. (2016). Epithelial-mesenchymal transition, IP3 receptors and ER-PM junctions: Translocation of Ca^2+^ signalling complexes and regulation of migration. Biochem. J..

[104] Franco S.J., Rodgers M.A., Perrin B.J., Han J., Bennin D.A., Critchley D.R., Huttenlocher A. (2004). Calpain-mediated proteolysis of talin regulates adhesion dynamics. Nat. Cell Biol..

[105] Casas-Rua V., Tomas-Martin P., Lopez-Guerrero A.M., Alvarez I.S., Pozo-Guisado E., Martin-Romero F.J. (2015). STIM1 phosphorylation triggered by epidermal growth factor mediates cell migration. Biochim. Biophys. Acta.

[106] Lopez-Guerrero A.M., Espinosa-Bermejo N., Sanchez-Lopez I., Macartney T., Pascual-Caro C., Orantos-Aguilera Y., Rodriguez-Ruiz L., Perez-Oliva A.B., Mulero V., Pozo-Guisado E. (2020). RAC1-Dependent ORAI1 Translocation to the Leading Edge Supports Lamellipodia Formation and Directional Persistence. Sci. Rep..

[107] Yu F., Hubrack S.Z., Chakraborty S., Sun L., Alcantara-Adap E., Kulkarni R., Billing A.M., Graumann J., Taylor C.W., Machaca K. (2019). Remodeling of ER-plasma membrane contact sites but not STIM1 phosphorylation inhibits Ca^2+^ influx in mitosis. Proc. Natl. Acad. Sci. USA.

[108] Samtleben S., Jaepel J., Fecher C., Andreska T., Rehberg M., Blum R. (2013). Direct imaging of ER calcium with targeted-esterase induced dye loading (TED). J. Vis. Exp..

[109] Berridge M.J., Bootman M.D., Roderick H.L. (2003). Calcium signalling: Dynamics, homeostasis and remodelling. Nat. Rev. Mol. Cell Biol..

[110] Bong A.H.L., Monteith G.R. (2018). Calcium signaling and the therapeutic targeting of cancer cells. Biochim. Biophys. Acta Mol. Cell Res..

[111] Roderick H.L., Cook S.J. (2008). Ca^2+^ signalling checkpoints in cancer: Remodelling Ca^2+^ for cancer cell proliferation and survival. Nat. Rev. Cancer.

[112] Monteith G.R., McAndrew D., Faddy H.M., Roberts-Thomson S.J. (2007). Calcium and cancer: Targeting Ca^2+^ transport. Nat. Rev. Cancer.

[113] Clark A.G., Vignjevic D.M. (2015). Modes of cancer cell invasion and the role of the microenvironment. Curr. Opin. Cell Biol..

[114] Dong H., Shim K.N., Li J.M., Estrema C., Ornelas T.A., Nguyen F., Liu S., Ramamoorthy S.L., Ho S., Carethers J.M. (2010). Molecular mechanisms underlying Ca^2+^-mediated motility of human pancreatic duct cells. Am. J. Physiol. Cell Physiol..

[115] Su L.T., Agapito M.A., Li M., Simonson W.T., Huttenlocher A., Habas R., Yue L., Runnels L.W. (2006). TRPM7 regulates cell adhesion by controlling the calcium-dependent protease calpain. J. Biol. Chem..

[116] Chen J.P., Luan Y., You C.X., Chen X.H., Luo R.C., Li R. (2010). TRPM7 regulates the migration of human nasopharyngeal carcinoma cell by mediating Ca^2+^ influx. Cell Calcium.

[117] Gao H., Chen X., Du X., Guan B., Liu Y., Zhang H. (2011). EGF enhances the migration of cancer cells by up-regulation of TRPM7. Cell Calcium.

[118] Rybarczyk P., Gautier M., Hague F., Dhennin-Duthille I., Chatelain D., Kerr-Conte J., Pattou F., Regimbeau J.M., Sevestre H., Ouadid-Ahidouch H. (2012). Transient receptor potential melastatin-related 7 channel is overexpressed in human pancreatic ductal adenocarcinomas and regulates human pancreatic cancer cell migration. Int. J. Cancer.

[119] Wondergem R., Ecay T.W., Mahieu F., Owsianik G., Nilius B. (2008). HGF/SF and menthol increase human glioblastoma cell calcium and migration. Biochem. Biophys. Res. Commun..

[120] Waning J., Vriens J., Owsianik G., Stüwe L., Mally S., Fabian A., Frippiat C., Nilius B., Schwab A. (2007). A novel function of capsaicin-sensitive TRPV1 channels: Involvement in cell migration. Cell Calcium.

[121] Monet M., Lehen’kyi V., Gackiere F., Firlej V., Vandenberghe M., Roudbaraki M., Gkika D., Pourtier A., Bidaux G., Slomianny C. (2010). Role of cationic channel TRPV2 in promoting prostate cancer migration and progression to androgen resistance. Cancer Res..

[122] Dhennin-Duthille I., Gautier M., Faouzi M., Guilbert A., Brevet M., Vaudry D., Ahidouch A., Sevestre H., Ouadid-Ahidouch H. (2011). High expression of transient receptor potential channels in human breast cancer epithelial cells and tissues: Correlation with pathological parameters. Cell Physiol. Biochem..

[123] Jardin I., Diez-Bello R., Lopez J.J., Redondo P.C., Salido G.M., Smani T., Rosado J.A. (2018). TRPC6 Channels Are Required for Proliferation, Migration and Invasion of Breast Cancer Cell Lines by Modulation of Orai1 and Orai3 Surface Exposure. Cancers.

[124] Monteith G.R., Davis F.M., Roberts-Thomson S.J. (2012). Calcium channels and pumps in cancer: Changes and consequences. J. Biol. Chem..

[125] Pera E., Kaemmerer E., Milevskiy M.J.G., Yapa K., O’Donnell J.S., Brown M.A., Simpson F., Peters A.A., Roberts-Thomson S.J., Monteith G.R. (2016). The voltage gated Ca^2+^-channel Cav3.2 and therapeutic responses in breast cancer. Cancer Cell Int..

[126] Kanwar N., Carmine-Simmen K., Nair R., Wang C., Moghadas-Jafari S., Blaser H., Tran-Thanh D., Wang D., Wang P., Wang J. (2020). Amplification of a calcium channel subunit CACNG4 increases breast cancer metastasis. EBioMedicine.

[127] Vashisht A., Trebak M., Motiani R.K. (2015). STIM and Orai proteins as novel targets for cancer therapy. A Review in the Theme: Cell and Molecular Processes in Cancer Metastasis. Am. J. Physiol. Cell Physiol..

[128] Jardin I., Rosado J.A. (2016). STIM and calcium channel complexes in cancer. Biochim. Biophys. Acta.

[129] Guan X. (2015). Cancer metastases: Challenges and opportunities. Acta Pharm. Sin. B.

[130] Chiang S.P., Cabrera R.M., Segall J.E. (2016). Tumor cell intravasation. Am. J. Physiol. Cell Physiol..

[131] Heerboth S., Housman G., Leary M., Longacre M., Byler S., Lapinska K., Willbanks A., Sarkar S. (2015). EMT and tumor metastasis. Clin. Transl. Med..

[132] Schwab A., Stock C. (2014). Ion channels and transporters in tumour cell migration and invasion. Philos. Trans. R. Soc. Lond. B Biol. Sci..

[133] Prevarskaya N., Ouadid-Ahidouch H., Skryma R., Shuba Y. (2014). Remodelling of Ca^2+^ transport in cancer: How it contributes to cancer hallmarks?. Philos. Trans. R. Soc. Lond. B Biol. Sci..

[134] Moccia F., Poletto V. (2015). May the remodeling of the Ca^2+^ toolkit in endothelial progenitor cells derived from cancer patients suggest alternative targets for anti-angiogenic treatment?. Biochim. Biophys. Acta.

[135] Zuccolo E., Bottino C., Diofano F., Poletto V., Codazzi A.C., Mannarino S., Campanelli R., Fois G., Marseglia G.L., Guerra G. (2016). Constitutive Store-Operated Ca^2+^ Entry Leads to Enhanced Nitric Oxide Production and Proliferation in Infantile Hemangioma-Derived Endothelial Colony-Forming Cells. Stem. Cells Dev..

[136] Xie J., Pan H., Yao J., Zhou Y., Han W. (2016). SOCE and cancer: Recent progress and new perspectives. Int. J. Cancer.

[137] Venkatachalam K., Van Rossum D.B., Patterson R.L., Ma H.T., Gill D.L. (2002). The cellular and molecular basis of store-operated calcium entry. Nat. Cell Biol..

[138] Flourakis M., Lehen’kyi V., Beck B., Raphael M., Vandenberghe M., Abeele F.V., Roudbaraki M., Lepage G., Mauroy B., Romanin C. (2010). Orai1 contributes to the establishment of an apoptosis-resistant phenotype in prostate cancer cells. Cell Death Dis..

[139] Hooper R., Zhang X., Webster M., Go C., Kedra J., Marchbank K., Gill D.L., Weeraratna A.T., Trebak M., Soboloff J. (2015). Novel Protein Kinase C-Mediated Control of Orai1 Function in Invasive Melanoma. Mol. Cell Biol..

[140] Yen M., Lewis R.S. (2019). Numbers count: How STIM and Orai stoichiometry affect store-operated calcium entry. Cell Calcium.

[141] Hodeify R., Selvaraj S., Wen J., Arredouani A., Hubrack S., Dib M., Al-Thani S.N., McGraw T., Machaca K. (2015). A STIM1-dependent ’trafficking trap’ mechanism regulates Orai1 plasma membrane residence and Ca^2+^ influx levels. J. Cell Sci..

[142] Hogan P.G., Lewis R.S., Rao A. (2010). Molecular basis of calcium signaling in lymphocytes: STIM and ORAI. Annu. Rev. Immunol..

